# Mutant p53 Protein and the Hippo Transducers YAP and TAZ: A Critical Oncogenic Node in Human Cancers

**DOI:** 10.3390/ijms18050961

**Published:** 2017-05-03

**Authors:** Maria Ferraiuolo, Lorena Verduci, Giovanni Blandino, Sabrina Strano

**Affiliations:** Molecular Chemoprevention and Oncogenomic and Epigenetic Units, Italian National Cancer Institute “Regina Elena”, 00144 Rome, Italy; maria.ferraiuolo@ifo.gov.it (M.F.); lorenaverduci@gmail.com (L.V.); giovanni.blandino@ifo.gov.it (G.B.)

**Keywords:** mutant p53, Hippo pathway, YAP, TAZ, tumor suppressor, oncogene, migration, proliferation, apoptosis, senescence

## Abstract

p53 protein is a well-known tumor suppressor factor that regulates cellular homeostasis. As it has several and key functions exerted, p53 is known as “the guardian of the genome” and either loss of function or gain of function mutations in the *TP53* coding protein sequence are involved in cancer onset and progression. The Hippo pathway is a key regulator of developmental and regenerative physiological processes but if deregulated can induce cell transformation and cancer progression. The p53 and Hippo pathways exert a plethora of fine-tuned functions that can apparently be in contrast with each other. In this review, we propose that the p53 status can affect the Hippo pathway function by switching its outputs from tumor suppressor to oncogenic activities. In detail, we discuss: (a) the oncogenic role of the protein complex mutant p53/YAP; (b) TAZ oncogenic activation mediated by mutant p53; (c) the therapeutic potential of targeting mutant p53 to impair YAP and TAZ oncogenic functions in human cancers.

## 1. Introduction

The p53 protein is a key factor for the safeguard of different cellular processes such as gene expression, cell cycle regulation, DNA damage repair, ageing, cell migration inhibition, and apoptosis. It was first identified in 1979 as a partner of the T antigen in SV40 viruses [1,2,3,4]. Therefore, p53 was primarily considered as a transforming factor until the 1980s, when it was definitively recognized as a tumor suppressor factor and named “the guardian of the genome”. *TP53* gene exhibits a high mutation rate in human tumors (about 50–70%) which results either in loss or gain of function of oncogenic mutant p53 proteins [5,6,7,8].

The Hippo pathway is evolutionarily conserved and tightly controls embryos and adult tissue organ size and growth. It is heavily involved in the control of proliferation, organ size and shape during development, stem cell maintenance, metastasis, tissue regeneration, apoptosis, senescence, and differentiation. It is regulated by many factors such as cell density and polarity, metabolism, senescence, and DNA damage [9,10,11,12]. Hippo crosstalks with other signaling players resembling a network rather than a linear pathway [13,14,15]. Dysregulations in the Hippo pathway play a role in cancer insurgence [9,16,17,18,19]. Recent evidences show that the p53 and Hippo pathways are “functionally” and “physically” connected; they regulate common pathways preserving cellular and tissue homeostasis in healthy conditions. When aberrantly deregulated, the p53 and Hippo pathways promote pathological and pro-tumorigenic onsets. Understanding this complex interaction could be of great interest in dissecting tumor onset and ultimately for the design of new anticancer strategies that could concomitantly target both pathways.

## 2. p53 and Mutant p53 Protein Functions

p53 is a short-lived protein (6–20 min half-life) that shuttles from cytoplasm to nucleus in response to different stresses that can erode the genome integrity (i.e., DNA damage, activation of oncogenes, hypoxia, viral replication or depletion of ribonucleotides) [3,20]. Moreover, it becomes activated by several post-translational modifications such as phosphorylation, sumoylation, acetylation and prolyl-isomerization mediated by physical interaction with diverse partners such as p300, PCAF, HIPK2, SUMO1, Chk1, Chk2, ATM, ATR, and Pin1 [21,22]. p53 is a modular protein harboring four functional different domains [23]: the N-terminal domain or transactivation domain (NTD or TA) that includes a proline-rich region and a transcriptional activation domain essential for binding to transcription factors and regulators of p53 activity [24,25]; the core domain that allows the binding to DNA (DBD domain) [26,27]; the oligomerization domain (OLD) relevant for the tetramerization of p53 [28] and the C-terminal domain or regulatory domain (RD) which is involved in post-translational modifications (phosphorylation, acetylation, ubiquitination, sumoylation, prolyl-isomerization, methylation, glycosylation, and ADP-ribosylation) which affect p53 activity [23].

### 2.1. Transcriptional Regulation

The most important biochemical activity of p53 is the ability to regulate gene transcription. Through its TA domain, p53 interacts with TBPs and TAFs inducing the recruitment of the RNA Polymerase II and consequently the transcriptional activation of its targets genes harboring a p53 responsive element in the DNA sequence (p53RE) [29]. p53 transcriptionally controls a few hundred genes involved in the regulation of cell proliferation, DNA damage repair, and apoptosis induction [30,31,32,33].

### 2.2. Cell Cycle Regulation

The p53 protein exerts an important role in regulating cell cycle checkpoints [34,35]. In particular, p53 induces *P21^WAF1/CIP1^* transcription that inhibits cyclin dependent kinases/cyclin complexes. This results in the activation of the G1 checkpoint [36,37,38]. Moreover, p53 also acts in the late G2 phase activating the DNA damage checkpoint [39]. When activated by stress or exogenously overexpressed, p53 can induce cell cycle arrest in G1 and G2/M phases and, inhibiting PCNA protein, p53 can induce cell replication blockage [3,40]. In response to DNA damage, ATM and ATR kinases phosphorylate p53, Chk2, Chk1, Mdm2, H2AFX, and BRCA1 inducing cell cycle arrest and promoting DNA damage repair [41,42,43,44,45]. G2/M arrest is mediated by p53-dependent *14-3-3σ* transcription, which inhibit Cdk1/cyclin B complexes [46]. Moreover, p53 regulates centrosome formation, mitotic spindle assembly, and chromosome segregation activating the spindle checkpoint [47,48] and interacts with the transmembrane protein Gas1 maintaining cells in the G0 phase [3,49].

### 2.3. DNA Damage Response

One of the main functions of p53 is the maintenance of genome integrity. It has been shown that different types of DNA damage, such as ionizing radiation, chemicals, radiomimetic drugs, ultraviolet radiation, and some pharmacological treatments can induce DNA damage [50]. When the insult is not efficiently repaired, changes in chromatin structure, genetic mutations and oncogenic transformation may occur [45]. These alterations in DNA sequence or structure activate p53, which in turn *trans*-activates different genes involved in cell cycle arrest, DNA damage repair or apoptosis [51,52]. If *TP53* is deleted or mutated, DNA damage will persist and cells replicate transmitting altered genetic heritage; this mechanism could lead to a neoplastic transformation. Moreover, p53 exerts other functions contributing to the maintenance of genome integrity: it exerts a 3′–5′ exonuclease activity [53] that is important in DNA recombination, in DNA repair and DNA replication [54]; p53 prevents chromosomic aberrations inhibiting aneuploidy and polyploidy occurrences; it blocks DNA replication until complete chromosome segregation [55]. p53 can also stimulate DNA damage repair activating GADD45 protein that can bind to PCNA and stimulate the NER system [56]; moreover, p53 can bind to TFIIH factor involved directly in the NER system [57,58].

### 2.4. Senescence and Aging

The p53 family members are involved in regulating ageing processes [23,59,60]. Mice models overexpressing wild-type p53 protein or p53 heterozygous mutants show reduced susceptibility to tumor formation and reduced lifespan [60,61] associated with premature aging [59,62]. p53 protein is responsible for irreversible cell cycle arrest, a characteristic of senescent cells [63,64]. In fact, *P16^INK4^* and *PML*, two key factors in senescence, are p53 transcriptional targets [65]. Normal cultured cells, subjected to increased number of cell divisions, display an increased p53 activity and enhanced p21 protein levels [3]. Therefore, following cell cycle arrest the p53 protein can activate two distinct pathways: senescence or apoptosis [62] that both can inhibit cell transformation and cancer onset [66].

### 2.5. Apoptosis

The most studied p53 function is the induction of apoptosis. The first evidence came from a seminal work of Oren’s lab, in which p53 reintroduction in myeloid leukemia cells-deficient for p53 promoted apoptosis [67,68]. Studies using transgenic and knockout mice demonstrated that endogenous p53 induced apoptosis, and thus counteracted tumorigenesis [69]. p53 protein acts as an apoptosis “facilitator” or “activator” [70] interacting through its DBD domain with anti-apoptotic (Bcl-2, Bcl-xL, Bcl-xW, and Mcl-1) [71] or with pro-apoptotic effectors (Bax, Bak, Bid, Bim, Bik, BMf, Bif-1, Bad, Noxa, and Hrk) inhibiting or activating them, respectively [70,72,73]. Puma protein is directly activated by p53 and can induce apoptosis binding to and inhibiting anti-apoptotic proteins [45,51,70]. p53 transcriptionally activates the pro-apoptotic genes *BAX* [74], *PUMA* [51] and *FAS-R* promoting apoptosis [45]. Moreover, p53 transcribes *IGF-BP3* coding Igf-bp3 protein that can bind to the growth factor IGF-1 and inhibits proliferation stimuli transduced by IGF-R receptors; p53 can also interact with WWOX inducing apoptosis synergistically [75]. Without growth factor stimuli, apoptosis pathway is activated [76]. Treatments with transcription or translation inhibitors such as Actinomycin D and Cycloeximide can induce p53-dependent apoptosis [2,77].

### 2.6. Metabolic Regulation

p53 plays a role in the regulation of cell metabolism. It has been shown that p53 regulates glucose uptake and glycolysis reducing the expression of glucose transporters (GLUT1, GLUT3, and GLUT4), phosphoglycerate mutase (PGM), and fructose-2,6-bisphosphate levels [78,79]; moreover, it induces the transcription of *TIGAR* and *GLS2* target genes [65,80,81] in both tumor and normal cell lines. It is also reported that p53 could induce some branches of the glycolytic pathway [82,83] and could regulate mitochondria metabolism [84,85,86,87,88]. In response to oxidative stress or DNA damage (induced by campothecin or danunorubicin treatments), p53 becomes activated and binds to the *GLS2* promoter inducing its transcription. This activation leads to increased glutamine metabolism and reduced ROS production [81]. It was proposed that Gls2 preserves total GSH levels with consequent reduction of oxidative stress and so promoting cell survival after mild genotoxic stress. Moreover, Gls2 exerts a tumor suppressor function representing a link between amino acid metabolism, ROS scavenging, and inhibition of tumor cells proliferation [81]. It is worthwhile to mention that p53 can conversely increase ROS production in response to severe stress conditions and thereby promote apoptosis [81]. Tigar protein encodes for a fructose bisphosphatase that down regulates cellular levels of fructose-2,6,-bisphosphate impairing the glycolytic pathway and leading to increased NADPH formation. High NADPH levels increase the GSH amount with consequent ROS detoxification [80]. p53 also regulates nucleic acid biosynthesis inactivating the glucose-6-phosphate dehydrogenase (G6PD) that is the rate-limiting enzyme of the pentose phosphate pathway (PPP) [89], and the lipid metabolism transcriptionally controlling many targets genes [90]. In detail, p53 can inhibit transcriptionally or by physical interaction some enzymes that promote lipid biosynthesis (SREBP-1 and G6PD) [89,91] or transcriptionally activate enzymes that inhibit lipogenesis promoting fatty acid oxidative metabolism (*SIRT1*, *Aromatase*, *Acad11*, *Lipin1*, *MCD*, and *DHRS3*) or also activate proteins that regulate cholesterol efflux (Caveolin 1) [92,93,94,95,96,97,98,99]. p53 family members p63 and p73 are also involved in cell metabolism supporting p53 tumor suppressor function [100].

p53 metabolic regulation appears crucial in the inhibition of cell transformation and tumorigenesis considering that tumor cells change their metabolism to support their rapid growth. In particular, tumor cells exhibit an increased glucose uptake, glycolysis, lactate production, glutamine consumption, nucleic acid metabolism, and lipid and cholesterol synthesis [90,101,102].

### 2.7. Cell Migration and Angiogenesis Inhibition

Cell migration is required for the acquisition of migratory and metastatic behavior in cancer cells. p53 protein regulates cellular expansion, cell polarity formation, and cell protrusion formation, which are required for cell motility [103]. The most critical event in tumorigenesis is the conversion from a primary tumor to an invasive and metastatic cancer. This transformation requires actin cytoskeleton remodeling with consequent changing in cell shape, and interactions among cells and extracellular matrix (ECM). Actin rich protrusions favor the interactions between cells and ECM and are regulated by Rho family members Rho, Rac, and Cdc42 [104]. Rho family members regulate epithelial to mesenchymal transition (EMT) [105] and a deregulation of these factors is involved in tumorigenesis and invasiveness [106,107,108,109,110]. p53 protein modulates the transcription of different factors involved in the regulation of cell motility and cell morphology: α-actin, collagen type IIa1 and VIa1, keratin, Msp, PAI1, HGF, VEGF, MMP-1, and fibronectin [111,112,113,114,115]. Moreover, p53 can interact with tubulin, vimentin, and F-actin suggesting a possible involvement in the regulation of microtubules, intermediate filaments, and microfilament formation [103]. p53 inhibits Rac, RhoA, and Cdc42 activation blocking filopodia formation, fibronectin formation, cellular expansion, and polarization essentials in promoting cell migration and invasion [116,117,118,119,120,121]. Moreover, p53 blocks Slug/Snail and EpCAM functions increasing E-cadherin levels, activates the tumor suppressor phosphatase PTEN [122,123], activates RhoGAPs and inhibits RhoGEFs with consequent inactivation of RhoA and ROCK kinase. Rho is also inhibited by Notch signaling activated by p53 [124].

p53 also exerts a key role in the negative regulation of angiogenesis activating a potent inhibitor of this process (TSP-1) and inhibiting the collagen α(II)PH protein involved in the growth of primary human endothelial cells [125,126].

As seen in all the functions previously described, p53 deregulation is primarily involved in cancer progressions instead of early tumorigenesis [127]. Indeed, loss of p53 function contributes to chemoresistance, increased invasiveness, and metastasis formation [128,129,130].

### 2.8. Mutant p53 and Cancer

The *TP53* coding gene is frequently mutated in human cancers (50–70% of cases) [131,132,133,134,135]. Most of these mutations are of missense type [135,136,137,138]. Missense mutations primarily affect the DBD domain (75% of cases) between exons 5–8 [6,139] and are roughly distinct in DNA-contact defective mutants (affecting the region involved in the binding to the DNA (i.e., p53-R275H and p53-R248W)) or in conformational mutants (affecting the tridimensional structure of the protein (i.e., p53-R175H and p53-R179H)) [26,132]. Most of the mutant p53 proteins do not recognize canonical consensus on the DNA sequence of wild-type p53 target genes [26,140] and exhibit an increased half-life (6–24 h) compared to that of wild-type p53 proteins. Heterozygous mutations in *TP53* denote increased cancer risk (around 90–95%) frequently in early ages [3]. *TP53* mutations are dominant negative and in heterozygous we can observe tetramers composed by native and mutant subunits that inactivate native ones abolishing p53 wild-type functions [6,141,142]. Mutant p53 proteins can also acquire new oncogenic functions (gain of function (GOF) mutations) promoting cell transformation, tumor progression, metastasis, and chemoresistance [5,6,7,143,144,145,146]. Moreover, GOF mutations hamper p53 proteasome-dependent degradation, altering the ordinary p53 turnover and promoting mutant p53 accumulation [141]. GOF of mutant p53 is due to different transcriptional activity caused by the recognition of new DNA secondary structures (i.e., MARs regions) or by the interaction with new protein partners and transcription factors [147,148,149]. GOF of mutant p53 proteins results also in the interaction with well-known tumor suppressor proteins (involved in the inhibition of cell proliferation, in apoptosis induction and differentiation) that are sequestered and inactivated (i.e., p73 and p63) [150,151,152,153,154,155]. It is known that mutant p53 binds with more affinity to p73 and p63 compared to the wild-type form and inhibits the *P21^WAF1/CIP1^* activation [150,151,156]. Knock-in mice with p53 missense mutations elegantly provided the proof that some mutant p53 proteins exert pro-tumorigenic activities [157]. 

#### 2.8.1. Mutant p53 Oncogenic Targets

Mutant p53 proteins show two critical residues in the N-terminal domain (TA), which are necessary for oncogenic function [158,159]. Different p53 mutants transcriptionally upregulate proto-oncogenes involved in nucleotide metabolism, amino acid and protein synthesis (different tRNA synthetases, *ASNS*, and *EEF1A2*), in cell metabolism (*IMPDH2*, *PHGDH*, *ALDH6A1*, and *AR*), in cell cycle progression (*CCNB2*, *CDC25A*, *cMyc*, and *MCL1*), in oncogenic transformation, cell migration and invasion (*RhoGAPs*, *RhoGEFs*, *ANGPT1*, *ITGA6*, *Zyxin 2*, *Fibronectin*, and *hEGR1*), in DNA replication and transcription (*POLD2* and *RNA pol II E*) and in anti-apoptotic signaling (*API5*, *E2F-5*, and *c-Fos*). All these mutant p53-dependend upregulated genes promote tumor transformation and progression in different cancer cell lines [160,161,162,163,164,165]. p53 GOF mutants are also involved in lipid metabolism increasing lipogenesis and cholesterol and fatty acid biosynthesis by the activation of SREBPs proteins [166]. It is still unknown if there is a specific DNA-binding consensus for mutant p53 (despite certain characterization of DNA consensus for wild-type p53), but it is clear that p53 mutants can bind to different transcription factors recruiting large protein complexes containing co-activators (SWI/SNF) or histones modifiers (KMT2A, KMT2D, and KAT6A), which cooperate with mutant p53 to activate or repress target genes [167,168].

Beside metabolic regulation that favors tumor transformation, p53 mutants can reinforce anti-apoptotic signaling downregulating the *MSP/MST-1* gene [169], or promote inflammation through the upregulation of interleukin 6 (*IL-6*) binding to and activating the transcription factor C/EBP β [170]. Moreover, mutant p53 promotes a pro-survival signaling inducing *HSP70*, *EGFR*, *hsMAD1*, *PCNA*, and *NF-κB* transcription and activation [171,172,173,174,175,176,177]. As mentioned before, mutant p53 activates target genes by binding to non-canonical partners that, in this specific case, are transcriptional factors that confer specificity to mutant p53 transcriptional activity. On that note, it is known that mutant p53 can interact with NF-Y transcription factor recruiting p300 acetyltransferase on the promoters of NF-Y target genes (*CCNA*, *CCNB1*, *CDK1*, and *CDC25C*) resulting in increased cell proliferation [178]. Moreover, interacting with Ets-1 transcription factor, mutant p53 activates the *MDR1* gene promoting chemoresistance [148]; alternatively, by binding to E2F-1 transcription factor, p53 mutants can activate *ID2* and *ID4* transcription promoting tumor neo-angiogenesis [179,180]. Binding to the E2F-4 transcription factor, mutant p53 represses *BRCA1* and *RAD17* genes impairing the DNA repair mechanism and promoting cancer genomic instability [181]. Mutant p53 is also recruited, together with p300, at the promoter of the proteasome activator *REGγ*, which promotes p53 wild-type, p21, and p16 protein degradation [182] and inhibits the expression of *KLF17* promoting cancer progression in invasive breast cancer cells [183]. Increasing evidence highlight the correlation between mutant p53 and increased cell metabolism associated with cancer progression. Freed-Pastor et al., demonstrated that mutant p53 protein cooperates with SREBPs proteins involved in fatty acid and sterol biosynthetic pathways; thereby leading to the upregulation of the mevalonate pathway that promotes survival and invasion of breast cancer cell lines [166]. In pancreas and breast cancer cell lines, mutant p53 can inhibit autophagy by the downregulation of BECN1, DRAM1, ATG12, SESN1/2, and P-AMPK and induce the activation of mTOR signaling promoting survival and cell proliferation [184]. It was also documented a mutant p53-dependent modulation of non-coding microRNAs (miRNAs) that contribute to cancer onset and progression [185,186,187,188].

#### 2.8.2. Mutant p53 Partners

Mutant p53 proteins are abundantly present in tumor specimens and cancer cells due to increased protein stability and nuclear localization [157,189]. They can interact with different partners determining the generation of large multi-protein floating complexes with oncogenic activities. Well-known mutant p53 partners are the p63 and p73 protein family members, which usually function as tumor suppressor genes cooperating with wild-type p53 in senescence, aging, and apoptosis induction [190,191,192]. Various mutant p53 proteins bind to p73 and p63 with high affinity compared to the wild-type form and this binding results in p73 and p63 sequestration and inactivation; thereby impairing senescence and apoptosis, and inducing chemoresistance in different cell lines [150,151,153,155,193,194,195,196,197]. Due to its high binding affinity to p73, mutant p53 disables the inhibitory complex p73/NF-Y promoting the oncogenic expression of *PDGFRb* in pancreatic cancer metastasis [198]. Similarly, mutant p53 sequesters p63 promoting TGFβ-induced metastasis [199]. Mutant p53 proteins not only transcriptionally activate proteins involved in lipids biogenesis, but also can interact with AMPK, inhibiting its kinase activity. How AMPK inhibition could contribute to increased lipid production, and thus tumor progression is still unclear [200]. It was reported that mutant p53, as an additional GOF activity, can interact with the nuclear matrix and MAR-DNA elements, generating chromatin domains that may enhance or repress transcription, and so perturb nuclear structure and function [147,149,201,202]. Valenti et al., showed that Plk2 protein binds to and phosphorylates mutant p53 leading to a more efficient recruitment of p300 on mutant p53 target genes with consequent enhanced cell proliferation and chemoresistance [203]. Interestingly, VDR physically and functionally interacts with mutant p53 and is able to convert vitamin D into an anti-apoptotic agent [204]. The prolyl-isomerase Pin1 amplifies mutant p53 oncogenic function co-activating a pro-aggressiveness transcriptional program in breast cancer [205]. Recent evidence demonstrate that two Bcl2 family proteins, BAG2 and BAG5, interact with mutant p53 preventing Mdm2 and CHIP-dependent proteasome degradation of mutant p53, this promotes mutant p53 protein accumulation and GOF determining tumor growth, cell migration, and chemoresistance [206,207].

## 3. The Hippo Pathway

Questions about developmental processes of multicellular organisms led to the discovery of four tumor suppressor genes in *Drosophila melanogaster*. Loss of function mutation in these genes results in overgrowth and reduced cell death [208,209,210]. These genes encode for the core components (also known as the core kinase cassette) of the Salvador-Warts-Hippo pathway and orthologous were discovered also in mammals. The Hippo pathway is evolutionary conserved and is involved in a plethora of physio- and pathological conditions [211].

### 3.1. Hippo Pathway Regulation, Regulators and Functions

**Figure 1:**
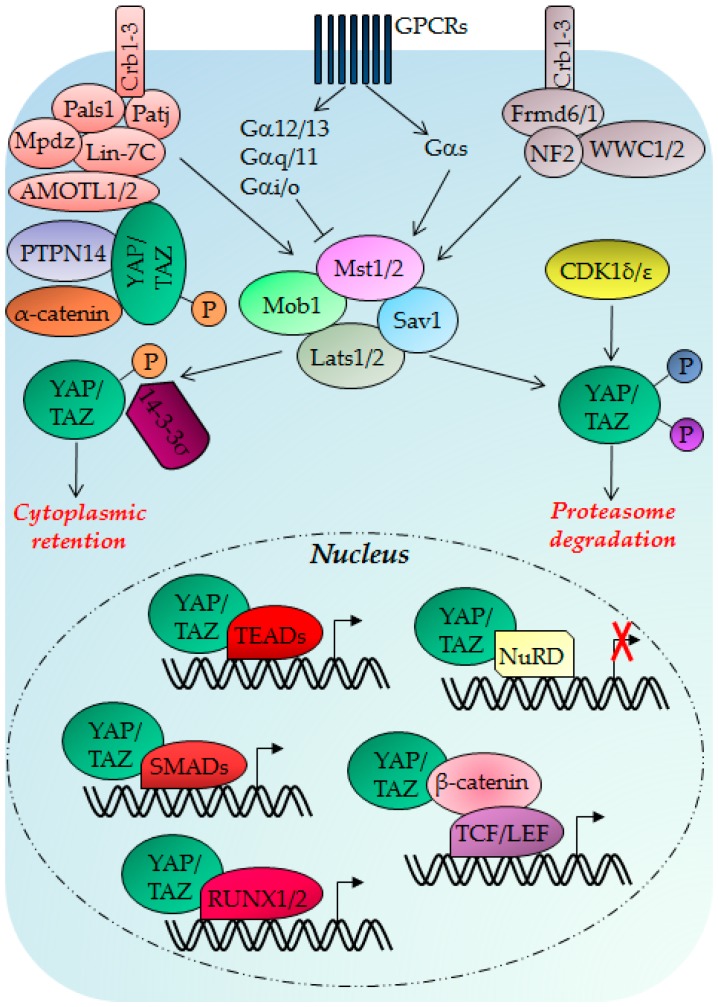
The Hippo pathway is activated by different stimuli and this activation induces Yes-Associated Protein (YAP) and Tafazzin (TAZ) phosphorylation and cytoplasmic retention. Moreover, the activated core kinase cassette (Mst1/2-Mob1-Sav1-Lats1/2) can contribute with CDK1δ/ε in inducing YAP and TAZ protein degradation. YAP and TAZ oncogenes, when activated and not phosphorylated, can translocate into the nucleus and bind to different transcription factors inducing osteoblast differentiation, cell proliferation, survival, mevalonate pathway activation, increased cell metabolism, anchorage-independent growth, EMT, ESCs and iPSCs pluripotency, cell migration, cell invasion, and tissue regeneration. Moreover, YAP and TAZ can function as co-repressors binding to the NuRD complex and inhibiting transcription of target genes. Arrows and T-bars indicate activating and inhibiting functions respectively. The symbol “X” in red color represents a transcriptional blockage.

Mst1/2, the adaptor protein Sav1 (or WW45), Lats1/2 AGC family kinases, and MOBKL1A/B (or Mob1) compose the mammalian Hippo kinase cassette [210,212]. Mst1/2 activate Lats1/2 by phosphorylation; Mob1 is necessary for both Lats1/2 and Mast1/2 activation and Sav1 is necessary for the Mst1/2 kinase activity [213]. This activated complex triggers a phosphorylation cascade that inhibits YAP and its paralog TAZ (or WWTR1) downstream effectors; these proteins are co-transcription factors that lack a DNA binding domain [214,215]. YAP/TAZ phosphorylation at Ser127 and Ser89, respectively, creates a 14-3-3σ binding site and consequent YAP/TAZ cytoplasmic retention and inhibition (Figure 1). Moreover, Lats1/2 can phosphorylate YAP and TAZ at Ser381 and Ser311 respectively, triggering ubiquitin-mediated proteasome degradation [216] (Figure 1). YAP exhibits two splicing variants (YAP1 and YAP2) displaying a different number of WW domains (one WW domain in YAP1 and two tandem WW domains in YAP2), one binding domain for the interaction with transcription factors, highly conserved PDZ-binding motifs, and an activation domain as for its paralog TAZ [215,217,218]. When activated, YAP and TAZ translocate in the nucleus and interact through the WW domain with the PPXY motifs of diverse transcription factors such as RUNX1 and RUNX2 regulating osteoblast differentiation of mesenchymal stem cells (MSCs), chondrocyte hypertrophy, endothelial cell migration and vascular invasion in bone development [218]. They also interact with Smads and TEADs/TEFs transcription factors, regulating cell proliferation, anchorage-independent growth, EMT, embryonic stem cells (ESCs) and induced pluripotent stem cells (iPSCs) [19,219,220]. YAP also interacts with β-catenin, inducing the expression of canonical Wnt target genes (*SOXS2* and *SNAI2*) [221] (Figure 1). TAZ interacts with PPAR-γ, TTF-1, Pax3, and Tbx5 inducing cell proliferation, cell migration, invasion, ESCs pluripotency and EMT [222,223]. Some YAP and TAZ targets are *CTGF*, *ANKRD1*, *CYR61*, *Birc2/cIAP1*, *Birc5*, *AREG*, and *cMyc* [214,224]. YAP/TAZ can function also as transcriptional co-repressors for the tumor suppressor genes *DDIT4* and *TRAIL* recruiting the NuRD deacetylase histones complex onto the promoters of selected genes [225] (Figure 1).

In contrast with conventional Lats1/2 tumor suppressor functions, it was recently demonstrated that, in different murine syngeneic tumor models (B16, SCC7, and 4T1), Lats1/2 exert an oncogenic function reducing tumor immunogenicity. In detail, Lats1/2 deletion induces interferon I response improving tumor vaccine efficacy; this mechanism opens possibilities in targeting Lats1/2 for cancer immunotherapy and suggests an additional function exerted by the Hippo pathway [226].

The NF2-Frmd6/1-WWC1/2 and Lin-7C/MMP5/Patj/Mpdz tumor suppressor complexes are involved in the mammalian apical-basal cell polarity (ABCP) and induce YAP/TAZ phosphorylation and inhibition [216,219]. AMOTL1/2, PTPN14, and α-catenin proteins maintain epithelial cell polarity and interact with YAP/TAZ sequestering them at the apical membrane and inhibiting their nuclear localization [227,228,229] (Figure 1). Several other proteins involved in ABCP integrity modulate the Hippo pathway [230]. In mechanical stretch conditions, YAP/TAZ activity is increased and is repressed when cells are compressed [10,231]. YAP/TAZ activation is also regulated by cell–cell contact abundance: high cell density induces YAP/TAZ phosphorylation and cytoplasmic retention [219]. It has been shown that GPCRs activate different heterotrimeric G proteins, which can activate or repress YAP/TAZ [232] (Figure 1). In response to stress-induced apoptosis, Mst1/2 are activated by auto-phosphorylation and caspase-dependent cleavage resulting in YAP phosphorylation and inactivation [233].

Organ size is finely regulated during development, and the Hippo signaling pathway plays a key role in this process [234]. YAP/TAZ are expressed during mammalian early embryogenesis and are crucial for inner cell mass (ICM) segregation from the trophectoderm (TE) in blastocyst development [230,234]. The Hippo pathway defines either differentiation or reprogramming to a pluripotent state that is determined by the precise balance of growth factor amounts and cytoskeletal-associated cues. YAP is found in the nucleus of MSCs, ESCs, and iPSCs; its nuclear localization and protein levels decline during MSCs and ESCs differentiation. Concomitantly, the Hippo pathway activity is enhanced and YAP accumulates in the cytoplasm of differentiated cells [224]. Increasing nuclear YAP/TAZ promotes tissue regeneration, rising cell proliferation in the liver, pancreas, salivary glands, kidney, lung, heart, intestine, skin, and nervous system [235].

The Hippo network is also a key player in the regulation of cell metabolism, and conversely is regulated by metabolic factors. It was shown in zebrafish models, that YAP upregulates nucleotide biosynthesis through the induction of glutamine synthetase. This promotes tissue growth during either development or tumorigenesis [236]. Mst1 kinase can inhibit glucose uptake reducing the expression of GLUT1 transporter and lactate production; this highlights the tumor suppressor function mediated by the Hippo core kinase cassette in regulating glucose metabolism [237]. The stearoyl-CoA-desaturase 1 (SCD1) enzyme promotes fatty acid synthesis and it is demonstrated to regulate YAP and TAZ function promoting their activation and nuclear localization that is also dependent by β-catenin activation. This promotes stemness, increased cell proliferation and it is associated with a poor prognosis in lung cancer casuistries [238]. Moreover, the phosphofructokinase (PFK1) enzyme that is involved in glycolytic metabolic pathway induces YAP and TAZ activity [239]; conversely, under glucose starvation conditions, Hippo and AMPK are activated leading to YAP phosphorylation and inhibition [240]. Hippo is regulated by the mevalonate pathway, as discussed below, and by TSC-mTOR pathway with consequent autophagy induction and protein synthesis inhibition [241]. Interestingly, the activated Hippo pathway controls cell metabolism acting as tumor suppressor factor; on the other hand, YAP and TAZ oncogenes induce increased cell metabolism promoting tumorigenesis and cancer progression.

### 3.2. Hippo Pathway Deregulation in Cancer

Down-regulation of the core kinase cassette components or activators of the Hippo pathway results in uncontrolled tissue overgrowth; moreover, YAP/TAZ overexpression induces cell proliferation, inflammation, acquisition of cancer stem cell features, EMT (forming metastases), inhibition of senescence, suppressed anoikis, reduced apoptosis, and drug resistance [9,16,18,213,216,230,242,243]. Generally, deregulation of the pathway promotes metastatic osteosarcomas, brain tumor development (meningiomas, schwannomas, and acoustic neuromas), hepatocellular carcinoma (HCC), bile duct tumors, and malignant mesothelioma [244]. YAP/TAZ genes are amplified and localized preferentially in the nucleus of several tumors: lung, pancreas, esophagus, gastric, skin, colon, prostate, liver, ovarian and mammary gland carcinomas, medulloblastomas, gliomas, and oral squamous-cell carcinomas [9,16,18]. Finally, the Hippo pathway interacts with many other signaling pathways, creating a complex network in which YAP/TAZ are emerging as a critical node integrating and decoding both oncogenic and tumor suppressor inputs [9,18,245]. 

Recently it was demonstrated that YAP could regulate non-coding RNAs biogenesis associated with cancer. In detail, at low cell density, nuclear YAP binds to and sequesters a regulatory protein of the miRNA-processing machinery, p72. This leads to widespread miRNA suppression in cells and tumors with concomitant cMyc post-transcriptional induction [246]. In lung cancer, YAP elicits its oncogenic activity sustaining the aberrant expression of the *MCM7* gene and its hosted miR-25, 93, and 106 cluster [247]. Moreover, nuclear YAP/TAZ elicits cytoplasm Dicer processing of pre-miRNAs [248]. In liver cancer, YAP/RUNX2 complex binds to the promoter of the tumor suppressor long non-coding (lncRNA) pseudogene *MT1DP* inhibiting its expression. Down-regulation of *MT1DP* enhances FoxA1 activity with consequent induction of the oncogenic factor *AFP*, a classic liver cancer biomarker [249]. It is also reported that YAP up-regulates lncRNA *MALAT1* expression at both transcriptional and post-transcriptional level facilitating proliferation and enhancing cell migration [250]. Another two lncRNAs have been investigated in colon and renal cancers. YAP cooperates with β-catenin in colon tumorigenesis, also activating the transcription of the lncRNA *RMRP* involved in ribosomal RNA processing [251]. The LncARSR and YAP axis forms a feed-forward loop in renal cancer. Forced expression of lncARSR expression enhances tumor renal initiating cells [252]. LncARSR, upregulated in renal tumor specimens is associated with a poor prognosis of renal cell carcinomas (RCCs).

## 4. *TP53* Status Impacts on the Hippo Pathway Activities

### 4.1. Wild Type p53 Protein and the Hippo Kinase Cassette in Tumor Suppression

It is known that the Hippo pathway and p53 act as tumor suppressors cooperating to induce senescence and apoptosis. In particular, for the Hippo pathway, this is mediated by the canonical function of inhibiting YAP and TAZ oncogenic activation, as previously described, and by additional non-canonical activities in which YAP functions as a tumor suppressor in response to stress conditions. 

**Figure 2:**
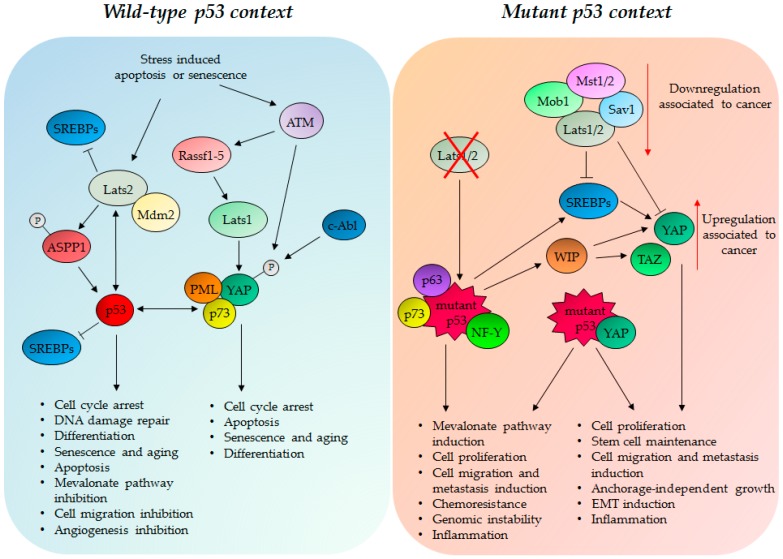
In a wild-type p53 context, DNA damage, oncogenic stress, senescence or apoptosis induction can trigger Lats1/2 activation and p53 and YAP mutual induction leading to a tumor suppressor response that is sustained by the p53/YAP/PML/p73 axis. Cancers harboring mutant p53 show an oncogenic cooperation between YAP/TAZ and mutant p53 that reinforce tumor growth, chemoresistance and migration in cancer cells. Restoring wild-type p53 functions or destroying oncogenic mutant p53/YAP/TAZ crosstalk could be good strategies for recovering the tumor suppressor program promoted by YAP and p53. Arrows and T-bars indicate activating and inhibiting functions respectively. The symbol “X” in red color represents a downregulation in protein levels.

Bai et al., demonstrate that HepG2 cells treated with diverse chemotherapeutics bear increased YAP nuclear accumulation that contributes to chemosensitivity [253]. In detail, nuclear YAP induces p21, Bax and Caspase 3 expression and inhibits the anti-apoptotic factors Bcl-2 and Bcl-xL. This results in cell cycle arrest and apoptosis induction that is p53-dependent as suggested by the binding of YAP to the p53 promoter [253]. YAP induces p53 transcription that in turn can bind to the promoter of YAP and activate YAP transcription in a positive feedback loop [253] (Figure 2). In this way, YAP and p53 sustain each other to induce apoptosis and chemosensitivity in hepatocellular carcinoma cells. Mitotic spindle checkpoint activation induces Lats2 activation and binding to Mdm2 protein with consequent p53 activation and G1/S arrest induction. In turn, p53 rapidly and selectively up-regulates Lats2 expression defining a positive feedback loop (Figure 2). The Lats2–Mdm2–p53 axis thus constitutes a novel spindle checkpoint pathway critical for the maintenance of proper chromosome number [254]. Lats2 is also demonstrated to explain a key role in the differentiation of ESCs in a p53-dependent manner [255]. In response to oncogenic stress, Lats2 phosphorylates the tumor suppressor protein ASPP1 and drives its translocation into the nucleus. Lats2 and ASPP1 lead p53 to pro-apoptotic promoters inducing death of polyploid cells [256] (Figure 2). Matallanas et al., demonstrated that the tumor suppressor proteins Rassf1-5 can interact to and activate Mst2 and Lats1 inducing cell cycle arrest and Fas-induced apoptosis [257]. In detail, in a Rassf1a overexpressing condition or activation of the death receptor by Fas ligand or in DNA damage conditions, ATM kinases activate Rassf1-5 proteins that in turns activate Lats1; this phosphorylates YAP protein in a different residue inducing nuclear localization and interaction with the p53 family member p73 [257] (Figure 2). YAP can stabilize p73 by preventing nuclear export and degradation, and together with p73 induces the transcription of pro-apoptotic genes such as *PUMA* and *p53AIP1* [257]. YAP functions as the transcriptional regulator of p73-mediated apoptosis, but is negatively regulated by a proto-oncogene Akt. Therefore, Akt phosphorylation of YAP Ser127, sequesters it in the cytoplasm and attenuates p73 apoptosis [258,259]. In response to DNA damage, YAP imparts transcriptional target specificity to p73, favouring its recruitment onto apoptotic, instead of growth suppression genes. The tumor suppressor *PML* gene is a target of the transcriptional competent complex YAP/p73; this leads to an auto-regulatory feedback loop that stabilizes YAP and maximizes p73’s pro-apoptotic activity [156,260,261,262] (Figure 2). Moreover, under DNA damage conditions, c-Abl kinase directly phosphorylates YAP at Tyr357, increasing protein stability and affinity for p73 [263] (Figure 2). YAP is also involved in the establishment of senescence through the activation of PML and p53 tumor suppressor proteins; in detail, loss of Werner-induced senescence activates ATM, which phosphorylates YAP and triggers the instauration of a transcriptionally active YAP/PML/p53 axis [12,264].

### 4.2. GOF Mutant p53 Protein, YAP and TAZ in Tumorigenesis

As described above, the cooperation between the wild-type p53 protein and the Hippo components can induce senescence, differentiation and apoptosis contrasting tumor transformation and progression. Interestingly, this scenario can be subverted when cells and tumors harbor mutant p53 protein with GOF activity. Indeed, it was recently documented that mutant p53 and YAP share a common transcriptional program showing a significant overlapping with gene signatures primarily involved in cell cycle regulation. Moreover, different GOF mutant p53 proteins (p53R280K, p53R175H, p53A193T, p53R248L, p53R273H, p53L194F, and p53P309S), but not wild-type p53 protein, can physically interact with YAP [265]. The protein complex YAP/mutant p53 can form with the transcription factor NF-Y a large multi-protein complex that is recruited onto the regulatory regions of *CCNA*, *CCNB*, and *CDK1* genes. This enhances gene transcription and leads to increased cell proliferation [265] (Figure 2). It was previously demonstrated that statins delocalize YAP in the cytoplasm thereby severely impairing its oncogenic activity [11]. In agreement with these findings, statins induce YAP cytoplasmic retention and consequently reduces the recruitment of mutant p53/NF-Y protein complex on the promoters of cell cycle regulated genes [265]. Since TAZ doesn’t appear to be involved in such a transcriptional network, at least in the reported experimental conditions, YAP may serve as a critical transducer of GOF mutant p53 proteins [265]. A more recent work highlights an oncogenic crosstalk involving mutant p53 proteins (harboring R175H and R273H missense mutations) and YAP/TAZ in glioma and breast cancer stem cells [266]. Mutant p53 proteins induce WASP-interacting protein (WIP) and promote the expression of the CSC-like markers CD133, CD44, YAP, and TAZ [266] (Figure 2). In detail, Akt2 whose activity is sustained by mutant p53/p63 oncogenic complex phosphorylates WIP. WIP phosphorylation induces YAP and TAZ activation and translocation into the nucleus with consequent activation of the YAP/TAZ oncogenic targets [266]. This work indicates that mutant p53 proteins can also cooperate with the TAZ protein [266]. Excess of cholesterol is associated with mammary tumor growth and represents an independent risk factor for breast cancer and for decreased response to endocrine therapies [267]. This is because some cholesterol metabolites induce proliferation of ER-positive breast cancer cells [268]. SREBP proteins are involved in cholesterol (mevalonate pathway), fatty acids, triglyceride, and phospholipid synthesis [269] and are frequently downregulated or overexpressed by tumor suppressor genes or oncogenes, respectively [252,267]. p53 and Lats2 cooperate to transcriptionally repress the expression of SREBPs (Figure 2); instead Lats2 downregulation induces SREBPs activation and overexpression of SREBP’s targets genes [252]. It was reported that Lats2 is crucial for cholesterol synthesis balance: Lats2 dysregulation is observed in some cases of nonalcoholic fatty liver disease (NAFLD). Lats2 restricts SBEBPs activity that is important in liver physiology and pathology. [252] This Lats2 activity is YAP-independent. Lats2 is necessary for p53 activation by cholesterol excess suggesting that the activation of the Lats2-p53 axis exerts a protective role in liver homeostasis [252,267]. On the other hand, when mutated, p53 increases SREBPs protein levels and transcription of their target genes (Figure 2). In this way, mutant p53 GOF increases the mevalonate pathway disrupting cell morphology and driving malignant phenotypes such as invasion [166]. Sorrentino et al., demonstrated that the mevalonate pathway, sustained by mutant p53 SREBPs activation increases YAP/TAZ oncogenic activity inducing cell proliferation and self-renewal in breast cancer cells [11] (Figure 2); moreover, YAP itself induces the transcription of several genes involved in cholesterol metabolism [250]. Mevalonate pathway inhibition, by treatment with statins, is able to reduce the oncogenic transcriptional effects of mutant p53 in a YAP-dependent manner, suggesting that the mevalonate pathway is both crucial as an upstream and downstream regulator of mutant p53 and YAP oncogenic functions [11]. p53 and Hippo crosstalk is also observed by Aylon et al., who demonstrated that Lats knockdown changes p53’s protein interactome and conformation, converting p53 tumor suppressor transcriptional program into ones activated in cancer-associated p53 mutants (Figure 2). This results in increased cell proliferation and migration [252]. 

p53 mutations, that result in the acquisition of new oncogenic functions, can change the interactome, conformation, and transcriptional program of wild-type p53 transforming the anti-oncogenic cooperation between Hippo and p53 in a pro-tumorigenic crosstalk primarily through the loss of Lats-p53 mutual activation and by the binding of mutant p53 to YAP (Figure 2). YAP exerts dualistic functions in response, to developmental stages, tumor transformation and stress stimuli. Indeed, it is functionally inhibited by the Hippo kinases but also activated by them in response to stress conditions. When activated, YAP promotes PML expression, p73- and p53-dependent senescence, differentiation, and apoptosis [12,260,264,270,271]. In a mutant p53 context, YAP is no more able to induce tumor suppressor responses but is bound to mutant p53, enhancing mutant p53 oncogenic functions with consequent increased cell proliferation, invasion, and chemoresistance in cancer cells (Figure 2). 

## 5. Conclusions and Future Perspectives

Since the crosstalk between the p53 and the Hippo tumor suppressor pathways can either elicit tumor suppressor or oncogenic effects, its therapeutic targeting might hold great potential for the treatment of human cancers. Indeed, p53 in its wild-type or mutant conformation and YAP/TAZ proteins represent suitable targets since the biological outputs of their interaction switch from pro-apoptotic activators (wild-type p53-p73-p63/YAP) to pro-tumorigenic and metastatic inducers (mutant p53/YAP/TAZ). Potentially, compounds that restore mutant p53 conformation to that of wild-type p53 protein might impair various oncogenic pathways promoted by mutant p53, also the oncogenic activity of the mutant p53/YAP protein complex and/or TAZ induction, and could reactivate the Hippo tumor suppressor function. A growing number of small molecules aimed to restore and stabilize the original DBD conformation of p53 have been identified. These include: p53 reactivation and induction of massive apoptosis (PRIMA-1), maleimide-derived molecule MIRA-1/NSC19630, short interfering mutant p53 peptides (SIMPs) (10–15 residues), reactivation of p53 and induction of tumor cell apoptosis (RITA), CP-31398 and many others [272,273,274]. These compounds showed great promise when tested in cancer cell lines, since they promote p53-induced apoptosis in tumor cells with mutant p53 proteins [275,276,277,278]. Despite its great selectivity and no side effects tested in cultured cell lines, PRIMA-1 does not reduce significantly tumor volume in in vivo experiments [277,279,280]. This result could be due to unknown potential secondary effects in in vivo mice models, to the higher time of treatment compared to the in vitro experiments and/or potential toxicity mediated by secondary products of PRIMA-1 metabolic degradation. Moreover, insulin-like receptors and others proto-oncogenes are responsible for resistance mechanisms to PRIMA-1 treatments [281]. Toxicity in actively proliferating cells is demonstrated after MIRA-1/NSC19630 treatments. The drug induces acute toxicity (within 2 h) in normal primary epithelial cells and in cancer cell lines inducing caspase-9-dependent apoptosis [282]. Cell toxicity is independent from p53 levels and mutational status; moreover, treated cells do not show resistance mechanisms in response to MIRA-1 treatments [282].

All these observations highlighted the need to develop new and more effective compounds and strategies, testing also chronic and acute potential toxicity in in vivo treatments. PRIMA-1MET (APR-246, developed by APREA), a compound very similar to PRIMA-1, but much more active at low dosage, restores the pro-apoptotic function of p53 with consequent activation of downstream target genes [275,283,284,285,286,287]. PRIMA-1MET has also successfully completed a Phase I clinical trial, showing a promising efficacy (ClinicalTrials.gov identifier: NCT00900614). Moreover, a small molecule, NSC59984 that restores wild-type p53 signaling in human tumors has been recently reported [288]. As mentioned before, statins can suppress mutant p53-dependent induction of cell proliferation through the inactivation of the YAP protein [11,265]. In a recent study, Parrales et al., demonstrated that statins can also induce degradation of conformational misfolded p53 mutants with minimal effects on wild-type p53. Thus, statins could preferentially suppress mutant p53 inhibiting cancer cell growth [289,290]. Drugs that inhibit YAP and TAZ functions (i.e., Verteporfin, LPA, thrombin, agonist for dopamine receptors, dobutamine, dasatinib, tankyrase inhibitors, and statins) have been reported; it appears that they mostly act indirectly on YAP/TAZ and exert their activity also inhibiting other targets; therefore they are not target-selective [244,291,292,293].

Further studies are necessary to elucidate mutant p53/Hippo crosstalk with the aim of designing specific combined therapies that could reactivate their pro-apoptotic function and disable their aberrant oncogenic interactions. In line with this, the elucidation of how p53 mutants and YAP interact each other, which domains are involved in this interaction, and if it is a direct or indirect interaction could be important to foster the identification of compounds aimed to destroy their cooperation. The targeting of the oncogenic mediators SREBPs and WIP proteins or the activation of Lats1/2 tumor suppression activities could be also very attractive. This might allow activating tumor suppressor pathways and impairing those with oncogenic outputs.

## Abbreviations

Acad11	Acyl-CoA Dehydrogenase Family Member 11
AFP	Alfa-fetoprotein
ALDH6A1	Aldehyde Dehydrogenase 6
α(II)PH	α(II) collagen Prolyl-4-Hydroxylase
AMOTL1/2	Angiomotin-like proteins 1 and 2
AMPK	AMP-activated Protein Kinase
ANGPT1	Angiopoietin 1
ANKRD1	Ankyrin Repeat Domain 1
API5	Apoptosis Inhibitor 5
AR	Aldose Reductase
AREG	Amphiregulin
ASNS	Asparagine Synthetase
ASPP1	Apoptosis-Stimulating Protein of p53 1
ATG12	Autophagy-related protein 12
ATM	Ataxia-Telangiectasia Mutated
ATP	Adenosine Triphosphate
ATR	ATM and Rad3-related protein kinase
Bad	Bcl-2-antagonist of cell death
BAG2	BCL2 Associated Athanogene 2
BAG5	BCL2 Associated Athanogene 5
Bax	Bcl-2-associated x protein
Bcl-2	B-cell lymphoma 2
Bcl-xL	B-cell lymphoma-extra Large
BECN1	Beclin-1
Birc2/cIAP1	Baculoviral Inhibitor of the apoptosis repeat-containing protein 1
Birc5	Baculoviral Inhibitor of the apoptosis repeat-containing protein 5
BRCA1	BReast CAncer type 1
CAMTA1	Calmodulin-binding Transcription Activator 1
CCNA	Cyclin A
CCNB	Cyclin B
Cdk1	Cyclin dependent kinase 1
CDK1	Cyclin Dependent Kinase 1
CHIP	Carboxyl terminus of Hsp70-Interacting Protein
Chk1	Checkpoint kinase homologue 1
Chk2	Checkpoint kinase homologue 2
CK1δ/ε	Casein Kinase 1 delta/epsilon
cMyc	V-Myc Avian Myelocytomatosis Viral Oncogene Homolog
CSCs	Cancer Stem Cells
CTGF	Connective Tissue Growth Factor
CYR61	Cysteine-Rich angiogenic inducer 61
DDIT4	DNA-Damage-Inducible Transcript 4
DRAM1	DNA Damage Regulated Autophagy Modulator 1
EEF1A2	Elongation factor 1 A-2
FoxA1	Forkhead box A1
Frmd6/1	FERM domain-containing protein 6 and 1
GADD45	Growth Arrest and DNA Damage gene 45
GLS2	Phosphate-activated mitochondrial glutaminase
GPCRs	G protein-coupled receptors
H2AFX	H2A histone Family member X
hEGR1	Early Growth Response protein 1
HGF	Hepatocyte Growth Factor
HIPK2	Homeodomain-Interacting Protein Kinase 2
hsMAD1	human Mitotic Arrest Deficiency protein 1
ID2	Inhibitor of DNA binding 2
ID4	Inhibitor of DNA binding 4
IGF-1	Insulin-Like Growth Factor-1
IMPDH2	Inosine-5′-Monophosphate Dehydrogenase 2
ITGA6	Integrin α-6
KATA6	Lysine Acetyltransferase 6A
KMT2A	Lysine Methyltransferase 2A
KMT2D	Lysine Methyltransferase 2D
Lats1/2	Large tumor suppressor 1 and 2
Lin-7C	Lin-7 homolog C
lncARSR	lncRNA Activated in RCC with Sunitinib Resistance, ENST00000424980
LPA	Lysophosphatidic Acid
MALAT1	Metastasis-Associated Lung Adenocarcinoma Transcript 1
MARs	Matrix Attachment Regions DNA elements
MCD	Malonyl-CoA Decarboxylase
MCL1	Human Myeloid Cell differentiation protein
Mdm2	Mouse double minute 2 homolog
MDR1	Multi Drug Reactivity 1
MMP-1	Matrix MetalloProteinase-1
MMP5	Membrane-associated Palmitoylated protein 5
MOBKL1A/B	Mps one binder kinase activator-like A and B
Mpdz	Multiple PDZ domain protein
Msp	Macrophage stimulator protein
Mst1/2	Mammalian Sterile-20 family Serine-Threonine kinases 1 and 2
MT1DP	Metallothionein 1D Pseudogene
NER	Nucleotide Excision Repair
NF2	Neurofibromin 2
NF-Y	Nuclear transcription Factor Y
p53AIP1	p53 Apoptosis Independent Protein 1
PAI1	Plasminogen stimulator Inhibitor 1
P-AMPK	Phospho-AMP-activated Protein Kinase
Patj	Pals1-associated TJ protein
Pax3	Paired box 3
PCAF	P300/CBP-Associated Factor
PCNA	Proliferating Cell Nuclear Antigen
PDGFRb	Platelet Derived Growth Factor Receptor b
PHGDH	3-Phosphoglycerate Dehydrogenase
Plk2	Polo-Like Kinase-2
PML	ProMyelocytic Leukemia
POLD2	DNA Polymerase Delta subunit 2
PPAR-γ	Peroxisome Proliferator-Activated Receptor γ
PTEN	Phosphatase and tensin homolog deleted on chromosome TEN
PTPN14	Tyrosine-Protein Phosphatase Non-receptor type 14
Puma	p53 upregulated modulator of apoptosis
Rassf	Ras-association domain family
RhoGAPs	Rho GTPase-Activating Proteins
RhoGEFs	Rho Guanine nucleotide Exchange Factors
RMRP	RNA component of Mitochondrial RNA Processing endoribonuclease
ROCK	Rho-associated protein Kinase
ROS	Reactive Oxygen Species
RUNX1	Runt-related transcription factor 1
RUNX2	Runt-related transcription factor 2
Sav1	Salvador 1
SESN1/2	Sestrin 1/2
SIRT1	Sirtuin 1
SNAI2	Snail family transcriptional Inhibitor 2
SOX2	Sex determining region Y-box 2
SREBPs	Sterol Regulatory Element Binding Proteins
TAFs	TBP Associated Factors
TAZ	Tafazzin
TBP	TATA-box Binding Proteins
Tbx5	T-box transcription factor 5
TCA	Tricarboxylic Acid
TEADs/TEFs	TEA Domain proteins/Transcription Enhancer Factors
TFIIH	Transcription Factor II Human
TIGAR	*TP53*-inducible glycolysis and apoptosis regulator
TRAIL	TNF-Related Apoptosis-inducing Ligand
TSC	Tuberous Sclerosis Complex 2
TSP-1	Thrombospondin-1
TTF-1	Thyroid Transcription Factor-1
VEGF	Vascular Endothelial Growth Factor
WASP	Wiskott Aldrich Syndrome Protein
WWC1/2	WW domain containing protein 1 and 2
WWOX	WW domain-containing Oxidoreductase 1
YAP	Yes-Associated Protein
